# Supplementary material for: Athletes’ Knowledge of Pelvic Floor Dysfunction and Their Knowledge of and Engagement with Pelvic Floor Muscle Training: A Scoping Review
Source: Int J Environ Res Public Health. 2025 Jan 14;22(1):104. doi: 10.3390/ijerph22010104 (PMC11764918; doi:10.3390/ijerph22010104)
Supplement: Supplementary file 1 [file ijerph-22-00104-s001.zip › Supporting Information Files_Sup 1.pdf]

<u>Supporting Information File 1:</u>

Search Strategies from all databases:

**Database**	**Search Number**	**PCC**	**Query**	**Results**
Scopus 3/1/24	3	Population AND Concept (limited to article title, abstract, keywords)	((athlete* OR sportswom?n OR sportsm?n OR sportsperson) AND ("Pelvic Floor" OR "urinary incontinence" OR "stress urinary incontinence" OR "urge urinary incontinence" OR "Fecal Incontinence" OR "pelvic organ prolapse" OR cystocele OR "rectal prolapse" OR "uterine prolapse" OR "visceral prolapse" OR "rectal prolapse" OR rectocele OR "Pelvic Floor Disorders" OR "Pelvic dysfunction" OR incontinen*))	294
19/12/23	2	Concept	"Pelvic Floor" OR "urinary incontinence" OR "stress urinary incontinence" OR "urge urinary incontinence" OR "Fecal Incontinence" OR "pelvic organ prolapse" OR cystocele OR "rectal prolapse" OR "uterine prolapse" OR "visceral prolapse" OR "rectal prolapse" OR rectocele OR "Pelvic Floor Disorders" OR "Pelvic dysfunction" OR incontinen*	205,300
19/12/23	1	Population	(Athlete* or sportswom?n or sportsm?n or sportsperson)	391,746

**Database**	**Search Number**	**PCC**	**Query**	**Results**
SportDiscus 23/12/23	3	Population AND Concept	(Athlete OR Sportswom?n OR Sportsm?n OR Sportsperson) AND "Pelvic floor" OR "urinary incontinence" OR "stress urinary incontinence" OR "urge urinary incontinence" OR "fecal incontinence" OR "pelvic organ prolapse" OR cystocele OR "rectal prolapse" OR rectocele OR "Pelvic	102

			Floor Disorders" OR "Pelvic dysfunction" OR incontinen*	
SportDiscus 23/12/23	2	Concept	"Pelvic floor" OR "urinary incontinence" OR "stress urinary incontinence" OR "urge urinary incontinence" OR "fecal incontinence" OR "pelvic organ prolapse" OR cystocele OR "rectal prolapse" OR rectocele OR "Pelvic Floor Disorders" OR "Pelvic dysfunction" OR incontinen*	1,051
SportDiscus 23/12/23	1	Population	Athlete OR Sportswom?n OR Sportsm?n OR Sportsperson	229,458

**Database**	**Search Number**	**PCC**	**Query**	**Results**
Web of Science 23/12/23	1	Population AND Concept	ALL=((Athlete OR Sportswom?n OR Sportsm?n OR Sportsperson) AND ("Pelvic floor" OR "urinary incontinence" OR "stress urinary incontinence" OR "urge urinary incontinence" OR "fecal incontinence" OR "pelvic organ prolapse" OR cystocele OR "rectal prolapse" OR rectocele OR "Pelvic Floor Disorders" OR "Pelvic dysfunction" OR incontinen*))	203

**Database**	**Search Number**	**PCC**	**Query**	**Results**
CINAHL Complete 23/12/23	1	Population AND Concept	((Athlete OR Sportswom?n OR Sportsm?n OR Sportsperson) AND ("Pelvic floor" OR "urinary incontinence" OR "stress urinary incontinence" OR "urge urinary incontinence" OR "fecal incontinence" OR "pelvic organ prolapse" OR cystocele OR "rectal prolapse" OR rectocele OR "Pelvic Floor Disorders" OR "Pelvic dysfunction" OR incontinen*))	156
