# Supplementary material for: Athletes’ Knowledge of Pelvic Floor Dysfunction and Their Knowledge of and Engagement with Pelvic Floor Muscle Training: A Scoping Review
Source: Int J Environ Res Public Health. 2025 Jan 14;22(1):104. doi: 10.3390/ijerph22010104 (PMC11764918; doi:10.3390/ijerph22010104)
Supplement: Supplementary file 1 [file ijerph-22-00104-s001.zip › Supporting Information Files_Sup 2.pdf]

Supporting Information File 2:

Athletes' Sports and Competition Levels

Study details - author/s, date	Competition level	No. of Different sports mentioned in each paper	Gymnastics, Dance and Cheerleading	Weightlifting and Gym Sports	Team Sports	Individual
Almousa, S & Van Loon, AB, 2019	Local, national and international	36	Trampolining, Dancing, Cheerleading, Pom pom dance, Gymnastics, Artistic gymnastics,	Weightlifting, Bootcamp/Crossfit,	Physical education students, Basketball, Volleyball, Field hockey, Softball, Handball, Soccer, Rowing	Tennis, Golf, Swimming, Track and field, Martial arts, Running, Walking, Water aerobics, Step aerobics, Rope jumping, Bike riding, Tennis, Cross country skiing, Wrestling, Judo, Boxing, Triathlon, Badminton, Pilates
Bo, K & Backe-Hansen, K. L., Norway, 2006	National	2			Soccer, Handball	
Bo, K & Nygaard IE, 2019	Local, National and International	10	Gymnastics, Trampolining	Crossfit, Powerlifting, Weightlifting	Volleyball, Soccer, Basketball	Running, Pilates,
Brennand, E et al. 2018	Not stated.	32		Crossfit, Interval training, HIIT, Bootcamp, T25, P90x, Kickboxing, Weight training, Strength training, Spin classes, Gym, Elliptical, Zumba,	Volleyball, Soccer, Curling, Baseball, Kickball	Running, Jogging, Treadmill, Yoga, Pilates, Stretching, Balance ball, Hiking, Walking, Stairs, Biking, Tennis, Skiing, Snowboarding
Campbell, KG et al., 2020	Not stated.	6		Impact classes		Running, Triathlon, Cycling, Swimming, Kayaking

Cardoso, A, Lima, C & Ferreira, C, 2018	Not stated.	6			Basketball, Volleyball, Handball, Futsal	Athletics, Judo
Carls, C, 2006	Not stated.	Not stated.				
Carls, C, 2007	Not stated.	7	Cheerleading, Pom pom dance	Weightlifting	Basketball, Softball, Volleyball	Track
Culleton-Quinn, E et al. 2022	Local, National and International	37	Artistic and rhythmic gymnastics, trampolining, cheerleading, dance, gymnastics, ballet, pom pom dance	Rope-skipping, weight lifting, bootcamp, CrossFit, powerlifting,	Volleyball, handball, basketball, futsal, softball, rugby, soccer, indoor football, badminton, field hockey, golf, hockey,	Swimming, Judo, athletics, track, track and field athletics, track athletics, tennis, cross country skiers, runners, karate, orienteering, Aerobics
de Souza Pereira, E, et al. 2022	Not stated	1		Crossfit		
Gan, ZS & Smith, AL, 2023	Not stated.	Not stated.				
Garrington, C, O''Shea, S & Pope, R 2022	Local	1			Volleyball	
Gill, N et al. 2017	Not stated.	1			Netball	
Gram, MCD & Bo, K 2020	National and International	1	Rhythmic gymnastics			
Hazar, HU 2020	Not stated.	1			Volleyball	
High et al. 2018	Not stated.	1		Crossfit		
Jacome, C et al. 2011	Local	6			Basketball, Indoor football	Sprinting, Middle-distance running, Long-distance running, and Jumping
Joseph, C et al. 2021	Not stated.	7	Trampolining, Gymnastics	Crossfit	Basketball, Volleyball, Handball	Triathlon

Krnicar, I, Scepanovic, D & Lukanovic, A 2004	Not stated.	1				Swimming
LÃºovÃksdÃ³ttir, I et al. 2018	Not stated.	11	Dance, Gymnastics,	Crossfit or Bootcamp together, Weightlifting	Handball, Football, Soccer	Running or jogging, Swimming, Cycling, Badminton
Mahoney, K, Heidel, RE & Olewinski, L, 2023	Not stated.	4		Weightlifting, Powerlifting, Strongman, Crossfit		
Moreno, TRP et al. 2022	International	28				
Neels, H et al. 2017	Local	Not stated.				
Parmigiano, TR et al. 2014	National and International	8			Basketball, Soccer, Handball	Track and field, Boxing, Judo, Wrestling, Swimming
Rohde, M et al. 2020	Not stated.	3		Weightlifting, Powerlifting, Crossfit		
Rolli, F & Frigeri, D 2016	Not stated.	1			Basketball	
Skaug, KL et al. 2022	National	3	Artistic gymnastics, Team gymnastics, Cheerleading			
Skaug, KL et al. 2022	National and International	2		Powerlifting and Olympic weightlifting		
Stickley, L & McDowell, D 2023	Local	10	Dance, Cheerleading, Gymnastics	Weightlifting	Soccer, Volleyball, Softball	Track and field/cross country, Waterskiing, Tennis
Thyssen, HH et al. 2002	National	8	Gymnastics, Ballet		Basketball, Volleyball, Handball, Badminton	Track athletics, Aerobics
Toprak Celenay, S, & Dusgun, ES, & Degirmendereli, AR 2021	Not stated.	3			Football, Basketball, Volleyball	

Wikander, L & Cross, D & Gahreman, DE 2019	Numerical grading?	1		Powerlifting		
Wikander, L et al. 2021	Local, National and International	1		Powerlifting		
Wikander, L et al. 2022	Local, National and International	1		Weightlifting		
Wikander, L, Kirshbaum, MN, Gahreman, DE, 2020	Local, National and International	1		Crossfit		
