# Supplementary material for: Athletes’ Knowledge of Pelvic Floor Dysfunction and Their Knowledge of and Engagement with Pelvic Floor Muscle Training: A Scoping Review
Source: Int J Environ Res Public Health. 2025 Jan 14;22(1):104. doi: 10.3390/ijerph22010104 (PMC11764918; doi:10.3390/ijerph22010104)
Supplement: Supplementary file 1 [file ijerph-22-00104-s001.zip › Supporting Information Files_Sup 3.pdf]

Supporting Information File S3:

Extracted definitions PFD:

Study details - author/s, date	PFD Extracted Definitions
Almousa, S & Van Loon, AB, 2019	UI, defined as the complaint of involuntary loss of urine. Including SUI (involuntary loss of urine on effort or physical exertion, or on sneezing or coughing) and UUI (the involuntary loss of urine associated with a sudden compelling desire to pass urine). Mentioned MUI.
Bo, K & Backe-Hansen, K. L., Norway, 2006	UI, defined as the complain of any involuntary leakage of urine including SUI (the complaint of involuntary leakage on effort or exertion, or on sneezing or coughing) and UUI (the complaint of involuntary leakage accompanied by or immediately preceded by urgency). Faecal incontinence, defined as loss of flatus, liquid or solid stool. Mixed incontinence mentioned.
Bo, K & Nygaard IE, 2019	UI, defined as the complaint of involuntary loss of urine during effort or physical exertion, or during sneezing or coughing. SUI, AI and POP were all mentioned but not defined.
Brennand, E et al. 2018	UI and SUI mentioned but not explicitly defined.
Campbell, KG et al., 2020	UI, defined as involuntary loss of urine including SUI (leakage associated with physical exertion such as sports, coughing and sneezing) and UUI (leaking associated with increased urgency or desire to void) and MUI (a combination of the symptoms of UUI and SUI).
Cardoso, A, Lima, C & Ferreira, C, 2018	UI, defined as any complaint of involuntary loss of urine including SUI, UUI and MUI.
Carls, C, 2006	Mentioned UI, SUI and UUI.
Carls, C, 2007	Mentioned UI, including SUI and UUI but not specifically defined.
Culleton-Quinn, E et al. 2022	Defined PFD as a collection of signs, symptoms and conditions that affect the pelvic floor, including UI, anorectal dysfunction, sexual dysfunction, pelvic organ prolapse and pelvic pain. Defined UI (the complaint of involuntary loss of urine) as the most common PFD, and included SUI (complaint of involuntary loss of urine on effort or physical exertion, or on sneezing or

	coughing) and UUI (complaint of involuntary loss of urine associated with urgency).
de Souza Pereira, E, et al. 2022	UI, defined as any complaint of involuntary loss of urine. SUI, defined as being directly related to physical exertion and an increase in IAP when it exceeds sphincter pressure. UUI and MUI were mentioned but not specifically defined.
Gan, ZS & Smith, AL, 2023	UI, including SUI and UUI but no specific definition given.
Garrington, C, O"Shea, S & Pope, R 2022	PFD encompasses a variety of conditions affecting the bladder, bowel and sexual function, such as incontinence and pelvic organ prolapse (POP). Including UI defined as involuntary urine leakage and AI, defined as involuntary leakage of gas, liquid or solid stool. POP refers to the descent of the bladder, uterus and/or rectum within the pelvis.
Gill, N et al. 2017	UI, including SUI and UUI. However, none of these terms were specifically defined.
Gram, MCD & Bo, K 2020	UI, defined as any complaint of involuntary leakage of urine. Including SUI (complaint of involuntary loss of urine on effort or physical exertion, or on sneezing or coughing). Mentioned UUI and MUI.
Hazar, HU 2020	UI, defined as the complaint of any involuntary leakage of urine.
High et al. 2018	Response of “yes” to “loss of stool you cannot control” defined FI. Response of “yes” to “Do you usually have bulge or something falling out that you can see or just feel in the vaginal area?” defined the presence of symptomatic POP. Responses of “moderate” or “quite a bit” for degree of bother from any urine leakage question defined UI. Subtypes of UI including UI with urgency or stress were evaluated by single-question responses. A report of at least moderate bother to the question “How much are you bothered by urine leakage with urgency (strong sensation of needing to go to the bathroom)?” defined urgency UI. A report of at least moderate bother to the question “How much are you bothered by urine leakage with laughing, coughing, or sneezing?” defined stress UI. If a positive response was given to at least 1 or those 2 urinary leakage questions, this was classified as general UI.

Jacome, C et al. 2011	UI, defined as an involuntary loss of urine. Mentioned SUI, UUI, and mixed UI.
Joseph, C et al. 2021	UI, defined as unintentional voiding of urine, which includes SUI (defined as occurring during situations of increased abdominal pressure such as coughing, lifting weights, exertion, sneezing, jumping, and squatting.
Krnicar, I, Scepanovic, D & Lukanovic, A 2004	SUI mentioned
LÃºovÃksdÃ³ttir, I et al. 2018	UI, defined as involuntary urination that is a social or hygienic problem and that is measurable. Including SUI (urination under physical stress e.g. coughing, laughing or exercising)
Mahoney, K, Heidel, RE & Olewinski, L, 2023	SUI, defined as involuntary leakage of urine from increased intra-abdominal pressure.
Moreno, TRP et al. 2022	UI, not specifically defined. SUI, defined as involuntary urine loss due to stress, coughing, or sneezing. Athletic UI, defined as loss related specifically to sports practice.
Neels, H et al. 2017	Included discussion of UI and SUI, but did not define either concept specifically.
Parmigiano, TR et al. 2014	UI mentioned, not defined.
Rohde, M et al. 2020	SUI, defined as a complaint of involuntary loss of urine during effort or physical exertion, or during sneezing or coughing.
Rolli, F & Frigeri, D 2016	Authors mentioned UI, including SUI and UUI. None of these terms were defined.
Skaug, KL et al. 2022	UI, not specifically defined. SUI, defined as the complaint of involuntary loss of urine on effort or physical exertion, or on sneezing or coughing. AI, defined as including involuntary loss of liquid or solid stool or gas.
Skaug, KL et al. 2022	Definitions of PFD was based on the International Urogynecological Association (IUGA)/International Continence Society (ICS) joint report on the terminology for female PFD. Includes UI, AI and POP. UI, defined as the complaint of involuntary loss of urine. SUI defined as the complaint of involuntary loss of urine on effort or physical exertion, or on

	sneezing or coughing. AI defined as the involuntary loss of faeces or flatus. POP defined as the downward descent of the female pelvic organs into or through the vagina.
Stickley, L & McDowell, D 2023	UI, defined as the complaint of any involuntary loss of urine. SUI, defined as the involuntary leakage of urine with physical exertion, sneezing, or coughing. UUI defined as urine leakage due to a sudden compelling desire to void that is difficult to prevent. MUI, defined as a combination of the symptoms of stress and urge UI.
Thyssen, HH et al. 2002	UI discussed, not specifically defined.
Toprak Celenay, S, & Dusgun, ES, & Degirmendereli, AR 2021	PFD includes UI (including SUI and UUI), AI, POP, lumbo-pelvic pain and sexual dysfunction. None of these terms were defined specifically.
Wikander, L & Cross, D & Gahreman, DE 2019	UI, defined as a complaint of involuntary loss of urine. SUI mentioned.
Wikander, L et al. 2021	UI, defined as a complaint of involuntary loss of urine. SUI, defined as complaint of involuntary loss or urine on effort or physical exertion including sporting activities, or on sneezing or coughing. UUI, defined as complaint of involuntary loss of urine associated with urgency. MUI, defined as complaint of both stress and urgency UI. Athletic incontinence, defined as UI experienced during athletic activity by otherwise continent women of any age or parity.
Wikander, L et al. 2022	UI, defined as a complaint of involuntary loss of urine. SUI, defined as complaint of involuntary loss of urine on effort or physical exertion including sporting activities, or on sneezing or coughing. UUI, defined as complaint of involuntary loss of urine associated with urgency. MUI, defined as complaint of both stress and urgency UI.
Wikander, L, Kirshbaum, MN, Gahreman, DE, 2020	UI and SUI mentioned. Athletic incontinence, defined as UI experienced during athletic activity or more specifically, UI experienced by otherwise continent young nulliparous women only during training and competition.
